# Identification of active signaling pathways by integrating gene expression and protein interaction data

**DOI:** 10.1186/s12918-018-0655-x

**Published:** 2018-12-31

**Authors:** Md Humayun Kabir, Ralph Patrick, Joshua W. K. Ho, Michael D. O’Connor

**Affiliations:** 1 School of Medicine, Western Sydney University, Campbelltown, NSW Australia; 2 Victor Chang Cardiac Research Institute, Darlinghurst, NSW Australia; 3 Department of Computer Science and Engineering, University of Rajshahi, Rajshahi, Bangladesh; 4 St. Vincent’s Clinical School, University of New South Wales, Sydney, NSW Australia; 5 Stem Cells Australia, Melbourne Brain Centre, University of Melbourne, Parkville, VIC 3010 Australia; 6 School of Biomedical Sciences, Li Ka Shing Faculty of Medicine, The University of Hong Kong, Pokfulam, Hong Kong, SAR China; 7 Molecular Medicine Research Group, Western Sydney University, Campbelltown, NSW Australia

**Keywords:** Signaling pathway, Gene expression, Protein-protein interaction, Dental epithelial cells, Lens epithelial cells, Lens fiber cells, Pluripotent stem cells, ROR1^+^ cells

## Abstract

**Background:**

Signaling pathways are the key biological mechanisms that transduce extracellular signals to affect transcription factor mediated gene regulation within cells. A number of computational methods have been developed to identify the topological structure of a specific signaling pathway using protein-protein interaction data, but they are not designed for identifying active signaling pathways in an unbiased manner. On the other hand, there are statistical methods based on gene sets or pathway data that can prioritize likely active signaling pathways, but they do not make full use of active pathway structure that link receptor, kinases and downstream transcription factors.

**Results:**

Here, we present a method to simultaneously predict the set of active signaling pathways, together with their pathway structure, by integrating protein-protein interaction network and gene expression data. We evaluated the capacity for our method to predict active signaling pathways for dental epithelial cells, ocular lens epithelial cells, human pluripotent stem cell-derived lens epithelial cells, and lens fiber cells. This analysis showed our approach could identify all the known active pathways that are associated with tooth formation and lens development.

**Conclusions:**

The results suggest that SPAGI can be a useful approach to identify the potential active signaling pathways given a gene expression profile. Our method is implemented as an open source R package, available via *https://github.com/VCCRI/SPAGI/*.

**Electronic supplementary material:**

The online version of this article (10.1186/s12918-018-0655-x) contains supplementary material, which is available to authorized users.

## Background

A key role cell signaling (also known as signal transduction) plays within biological systems is to relay extracellular signals in order to regulate intracellular gene expression. The signal transduction process is typically initiated by the binding of a ligand to a membrane-bound receptor, which triggers a cascade of intercellular signaling activities through multiple kinases - ultimately impacting on how transcription factors regulate downstream gene expression [1]. The coordinated activity of different signaling pathways within and between multiple cell types is the basis of many important biological processes, such as development, tissue repair and immunity [2, 3].

Activation of different signaling pathways can lead to numerous physiological or cellular responses, such as cell proliferation, death, differentiation, and metabolism [4, 5]. Any interruption that occurs within these extra−/intra-cellular communication chains can cause diseases including developmental disorders and cancers [6–9]. Conversely, a clear understanding of the activity of, and interaction between, signaling pathways can help to design rational disease treatment and tissue regeneration strategies [10]. It is therefore important to understand the signaling pathways that are activated in a cell, in order to provide a framework for understanding critical pathways affected by disease.

In principle, it should be possible to identify the important signaling pathways of a cell by using gene expression and protein-protein interaction (PPI) data sets. Extensive, publically-available PPI data provide an opportunity to establish a general signaling pathway blueprint, to which cell type-specific gene expression data can be mapped so as to refine the general signaling pathway blueprint into a cell-type specific blueprint. In this way it should be possible to construct a set of cell-type specific active signaling pathways for any cell that summarizes the information flow from a receptor (R) to kinases (Ks), then to transcription factors (TFs).

PPI data is a direct source of information about the structure of signaling pathways [3, 11]. A number of PPI databases are available for human and model organisms such as STRING [12]. A number of bioinformatics methods have been proposed for the reconstruction of known signaling pathways by using PPI data. For example, *CASCADE_SCAN* generates a specific pathway for a list of protein molecules using a steepest descent method. That is, the method takes the input proteins and then finds their interaction partners iteratively based on some evidences (i.e., high scored interactions) [1]. On the other hand, *Pathlinker* reconstructs the known signaling pathways by taking a subnetwork of PPI that consists of the Rs and TFs of interest [13]. The *PathLinker App* is a software tool of the *Pathlinker* method implemented as a *Cytoscape app* [14]. *PathFinder* identifies signaling pathways from a specific R protein to a TF protein in PPI networks by extracting the characteristics of known signal transduction pathways and their functional annotations in the form of association rules [15].

A number of methods use PPI data alone to infer signaling pathway structure. Gitter et al. proposed a method to handle the orientation problem in weighted protein interaction graphs as an optimization problem and present three approximation algorithms based on either weighted Boolean satisfiability solvers or probabilistic assignments [16]. Mei et al. proposed a multi-label multi-instance transfer learning method to simultaneously reconstruct 27 human known signaling pathways, and model their cross-talk [17]. Scott et al. proposed a method to reconstruct the known signaling pathways efficiently in protein interaction networks by assigning well-founded reliability scores to PPI data and by applying a color coding algorithm [18].

There are also methods that combine PPI and genetic interaction data to identify signaling pathway structure. The activity pathway network (APN) approach utilizes high-throughput genetic interaction data and applies the Bayesian learning method to identify detailed structure of known signaling pathways [19]. Another method utilizes the same approach to restructure the pathway by also combining PPI data with genetic interaction data [20].

A number of computational methods utilize PPI data along with gene expression data to uncover known signaling pathways [2, 3, 21, 22]. In these methods the gene expression data sets are usually used to calculate the edge weight by gene expression correlation for the network. One approach utilizes PPI and gene expression data sets and applies integer linear programming to get an optimal subnetwork from the PPI network starting from membrane proteins and ending at transcription factors [3]. A recently published method called *HISP* uses the same approach, but in addition applies genetic algorithms with operations including selection, crossover, and mutation to select the candidate topologies of resultant signaling pathways and uses gene knockout data to get directionality of the signaling pathways [2]. *Netsearch* determines networks by integrating protein-protein interaction data with microarray expression data by extracting subnetworks of the protein interaction dataset whose members have the most correlated expression profiles [22]. It generates a specific pathway based on the input proteins (R and TF) and the PPI networks. Another method highlights the order of signaling pathway components, assuming all the components on the pathways are known [21]. It constructs a score function based on the correlations between each gene pair to determine the final signal transduction network.

All of the above methods aim to restructure the topologies of known signaling pathways. However, to our knowledge, no open-source methods have been reported that simultaneously and comprehensively identify the set of active signaling pathways *and* the likely pathway structures for a gene expression profile (i.e., all R, K and effector TF paths for each identified pathway). Additionally, most of the above methods were evaluated and applied to yeast PPI data, with only a few methods designed specifically to deal with the significantly greater complexity of mammalian data. Here we propose an approach to systematically identify the set of active receptor-mediated signaling pathways within any given cell, by combining PPI and gene expression data. This method is implemented as an open source packaging using the ‘R’ programming language. This open source software is called SPAGI (**S**ignaling **P**athway **A**nalysis for putative **G**ene regulatory network **I**dentification), and is available via https://github.com/VCCRI/SPAGI/.

## Methods

### Building background pathway data

**Fig. 1:**
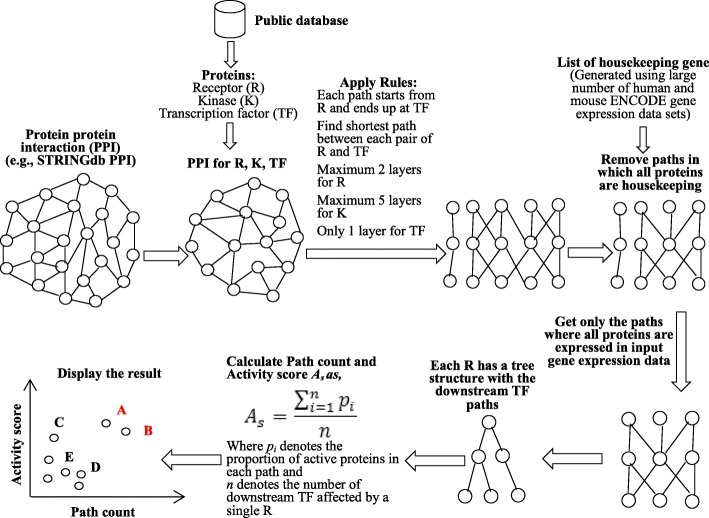
The workflow diagram of the SPAGI method

The overall workflow of the SPAGI approach is approach is depicted in Fig. 1. First we collected the known R, K and TF signaling molecules (2134 genes/proteins in total) from public data sets [23–25]. The list of R proteins was collected from a curated database of the Fantom5 project [24]. The list of K proteins was collected from the Uniprot curated database [23]. The list of TF proteins was obtained from a database of sequence-specific DNA-binding TFs identified by gene ontology (GO) based annotation [25]. Next we separately used both the mouse and human PPI data from *STRING* database (version 10) [26] to obtain all currently known PPIs for the 2134 known R/K/TF signaling molecules - while keeping the human and mouse separate. Please note that we have considered here all the physical and other inferred (e.g., co-expression) interactions when defining PPIs to maximize our ability to detect the full network structure. The confidence (*combined_score*) values assigned to interactions within STRING range from 0 to 999. We selected PPIs defined by STRING as ‘high confidence’ (i.e. *confidence_score > = 700*) to further maximise our ability to construct networks representative of true biological pathways. This thresholding yielded 16,550 and 19,502 PPIs for mouse and human respectively. After obtaining these highly scored PPIs both for the human and mouse organisms we have merged all the PPIs by assuming that the molecules have one-to-one homology mapping between the organisms. Note that after filtering and considering the presence of bi-directional interactions within *STRING* (e.g., R to K and K to R), the set of all known R/K/TF interactions involves 39,004 PPIs in human and 33,100 PPIs in mouse (with 27,790 PPIs common to both). We then took the union of all PPIs and have assigned the larger score value of a PPI if it is present in both organisms. The merged PPI network has 44,314 edges (See Table 1 for details).

**Table 1:** SPAGI pathway path background data summary

	**For Mouse**	**For Human**
# R, K, TF	2134	2134
# R/K, K/K, K/TF interactions (combined_score > 0)	234,603	249,571
# high-confidence (combined_score > = 700) R/K, K/K, K/TF interactions (assuming bi-directional interaction)	33,100	39,004
# common interaction	27,790	
# combined unique interaction	44,314	
# high-confidence complete R/K/TF paths	102,842	
# high-confidence complete R/K/TF paths without housekeeping gene paths (# of R-defined pathways)	89,161 (548)	

From the combined high scored PPIs, we collected only the PPIs for the signaling pathways that have interactions able to make full paths from R to K to TF. This process included interactions from:

- R (not directly connected with K) to R (directly connected with K)
- R to K
- K to K and
- K to TF

Finally, we collected all the filtered PPIs from the above step, keeping their associated PPI *combined_score* value for each of the interactions. Note that for clarity, the word ‘path’ is defined as a single R/K/TF prediction, whereas the word ‘pathway’ is defined as the collection of paths that all start from the same R (i.e., all paths defined by a single R constitutes a pathway).

To make the signaling pathway paths, we first made a directed weighted graph from the PPI data using the *igraph R* package. As *igraph* considers the weight of the interaction as a cost (i.e., higher weight means it needs more effort to travel), we have modified the PPI *combined_score* value as weight by calculating *1000-combined_score*. After assigning the *combined_score* as weight we collected the reachable shortest paths from each R protein to each TF protein by utilizing the *shortest_path* function of the package. The *shortest_path* function uses the Dijkstra’s graph algorithm for the weighted directed graph. We have collected all the complete paths (a path is being called complete if it starts from a R protein and ends up to a TF protein) that have a length from 3 to 7, allowing for at most 2 layers for RP, 5 layers for KN and 1 layer for TF. To identify cell type-specific paths, we then filtered out the complete paths where all factors were designated as housekeeping genes (see the next section for how the list of housekeeping genes was generated). As a result of these steps, the final collection of complete paths consists only of those that are not designated as housekeeping paths. These paths are used as background pathway path data for our method.

### Housekeeping genes identification

We collected the published RNA-seq gene expression data sets for different cells and tissues both for mouse and human from the ENCODE project [27, 28], and processed them separately. We examined the expression distribution pattern of these data sets and found that on average the *log2 (FPKM + 1) = 1.5* value could be used as the expression cut-off for the data sets. Using this cut-off we identified the expressed genes for all the cells and tissues. We then designated a gene as a housekeeping gene if it was found to be expressed in at least 75% of the total number of cells and tissues for that particular organism. This approach was used to identify both the mouse and human housekeeping genes. These 2 lists of housekeeping genes were then combined to generate a unique list of housekeeping genes, assuming one-to-one homology mapping between human and mouse genes. This combined list of unique housekeeping genes was used as background data.

### Potential signaling pathway identification

The background signaling pathway path data was used to identify the potential signaling pathways for a particular gene expression data set. As input we took the gene expression data matrix of *log2* transformation of *RPKM/FPKM/CPM* values, an expression cut-off threshold to identify the expressed genes, and a high expression threshold (generally an expression value greater that the expression value of the peak of distribution) to calculate the activity score of the pathways.

Processing steps

1. From the gene expression data set, first we calculated the average expression value of the replicates and then identified the expressed genes by using the cut-off threshold described above.
2. From the background path data we obtained only those paths for which all the protein factors are expressed according to the input gene expression data. This set of paths is treated as potential signaling pathway paths for the gene expression data set.

### Ranking of the potential signaling pathways

For each potential signaling pathway, we first calculated the proportion of active molecules (defined as highly expressed genes based on the above high expression threshold) for each path. We then summed all the proportions of all the paths for the pathway and divided the total proportion value by the total number of paths of the pathway. This final value was termed the *Activity score* (*A*_*s*_) for a pathway and mathematically can be written as:

$$ {\boldsymbol{A}}_{\boldsymbol{s}}=\frac{\sum_{\boldsymbol{i}=\mathbf{1}}^{\boldsymbol{n}}{\boldsymbol{p}}_{\boldsymbol{i}}}{\boldsymbol{n}} $$

Where *p*_*i*_ denotes the proportion of active molecules in each path and *n* denotes the number of downstream TFs for the pathway. Next we plotted the values of *n* and *A*_*s*_ to display the results of top ranked active signaling pathways in the upper positions.

### Assessment of SPAGI false positive rate

The SPAGI false positive rate was obtained by randomly assigning gene expression data and then re-performed the SPAGI analyses. The number of highly ranked active pathways for each sample was then counted. The false positive rate for highly ranked pathways was obtained by dividing the number of highly ranked pathways obtained from the randomly assigned data by the number of highly ranked pathways obtained from the original sample. GO analysis was also performed on the randomly assigned gene expression data used to determine the SPAGI false discovery rate. Each GO analysis was performed separately using the online version of Enrichr for biological processes [29]. Results were filtered to retain only the significant terms and for signaling GO terms using the raw *p*-value. The false positive rate for the GO analysis was calculated by dividing the number of highly ranked pathways obtained via the randomly assigned data by the number of highly ranked pathways obtained from the original data.

## Results

The ability of SPAGI to identify known, critical, tissue-specific signaling pathways was tested using four cell types obtained from three different gene expression data sets (two are RNA-seq and one is microarray). These four cell types were chosen as there is an extensive body of literature for them that has already identified critical pathways, thus enabling biological validation of the SPAGI output. The first data set used is from mouse dental epithelial cells at the development stage E13.5 (*n* = 3) [30]. The remaining two data sets were from the ocular lens: one is a newborn mouse lens data set that consists of gene expression profiles from lens epithelial cells (LECs; n = 3) and lens fiber (LF) cells (n = 3) [31]; the other data set is from human pluripotent stem cell-derived ROR1^+^ LEC–like cells (*n* = 2) [32].

### SPAGI analysis of tooth

**Fig. 2:**
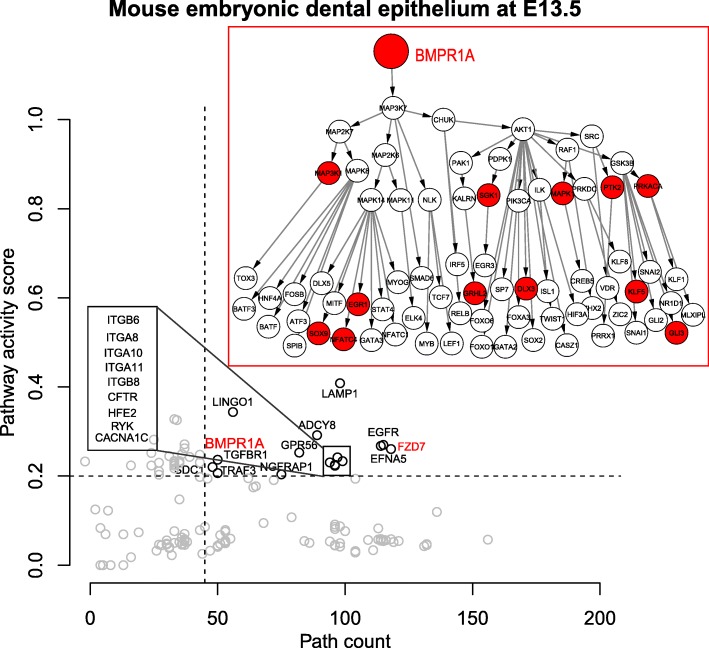
The result of mouse embryonic dental epithelium cell at E13.5. The red color indicates the known pathways. The details of BMPR1A pathway is shown in the figure

Published data have shown that BMP and WNT (through FZD receptors) signaling pathways are important for embryonic mouse tooth development [30]. Loss of function of BMPR1A in dental epithelial cells reduces WNT expression and prevents tooth formation [30]. To test whether SPAGI can identify BMP and WNT/FZD pathways from published dental epithelial cell gene expression data, we applied the SPAGI method to gene expression data from embryonic development stage E13.5. After all filtering, we have obtained 14,657 specific paths (i.e., 14.25% of total paths) for the dental epithelial cell. This analysis revealed SPAGI identified both the BMPR1A and FZD7 receptor-mediated pathways (Fig. 2), together with a range of other pathways.

### SPAGI analysis of lens gene expression data

A large number of studies over decades have described the requirement for different signaling pathways during lens development. As summarized in a recent review by Cvekl and Zhang [33], while critical lens pathways have been broadly identified, the precise R/K/TF signaling paths utilized within each pathway are not fully understood, nor are the path nodes (typically Ks and TFs) where different signaling pathways intersect. An accurate method for comprehensively identifying R/K/TF paths that operate within lens (and other) tissue is therefore needed. For example, the FGF pathway induces the pre-placodal region required for lens formation, as well as subsequent proliferation of LECs and differentiation of LECs to LF cells. The BMP pathway is also involved in pre-placodal induction, invagination of the lens placode, LEC proliferation and survival, and LF cell differentiation. The FZD pathway works as an inhibitor at the pre-placodal region, and in LEC adhesion, integrity and polarity. NOTCH signaling controls lens growth and acts as a differentiator for both LECs and LF cells. Signaling through different integrins is required early in lens differentiation, and for cell adhesion, lens capsule assembly and normal development of both LECs and LFs. Cadherins are required for appropriate polarity, adhesion and survival of LECs, and for LF cell elongation. EPHs and Ephrins are involved in cell adhesion and polarity, and LF cell elongation and alignment. The TGFβ pathway acts as an inhibitory signal in the pre-placodal region for proper lens growth, and is implicated in lens diseases such as anterior subcapsular cataract and posterior capsule opacification. Critically, how molecular integration of all these pathways occurs during lens development or formation of different cataract subtypes is currently unclear [34].

**Fig. 3:**
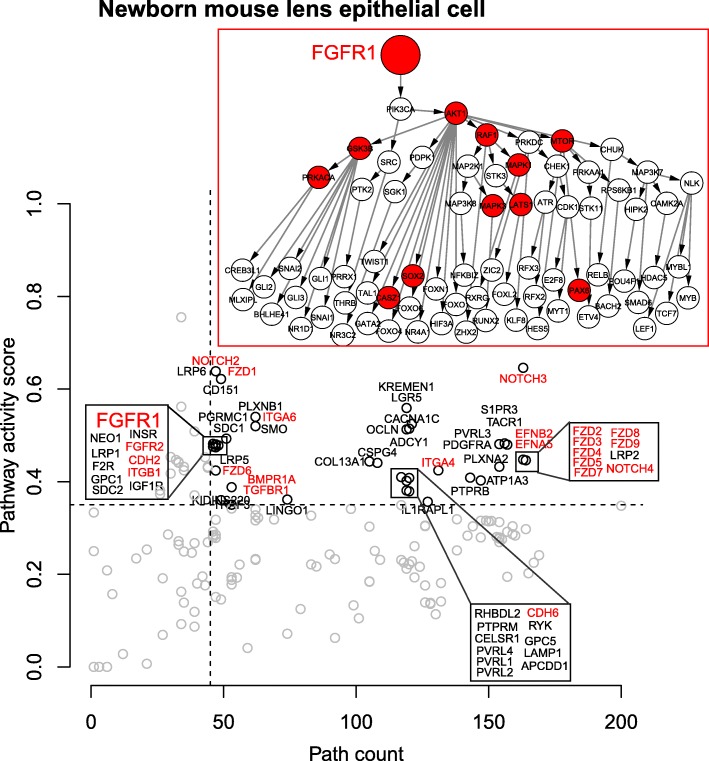
The result of newborn mouse lens epithelial cell. The red color indicates the known pathways. The details of FGFR1 pathway is shown in the figure

**Fig. 4:**
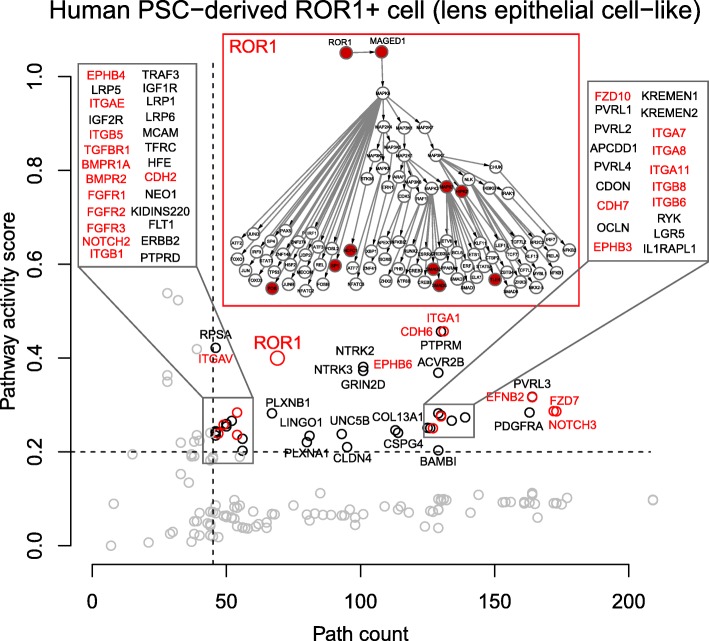
The result of human PSC-derived ROR1+ cell (lens epithelial cell-like). The red color indicates the known pathways. The details of ROR1 pathway is shown in the figure

Analysis of published mouse LEC gene expression data [31] using our SPAGI method identified all of the pathways mentioned above (Fig. 3). After all filtering this analysis gave us 25,624 specific paths (i.e., 24.92% of total paths) for the mouse LEC. Moreover, the activity score with the number of downstream TFs was able to preferentially rank these known critical lens pathways over other pathways identified within the mouse LEC data. Analysis of a large-scale source of human LECs (ROR1^+^ cells) [32] similarly identified the known lens signaling pathways (Fig. 4). This analysis also gave us 23,665 specific paths (i.e., 23.01% of total paths) for ROR1^+^ cell after all filtering.

**Fig. 5:**
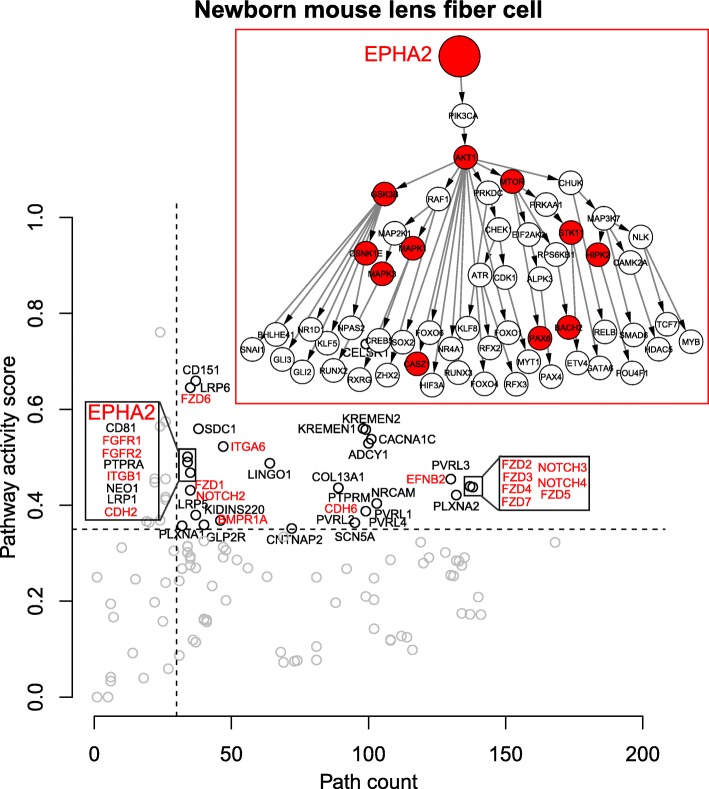
The result of mouse lens fiber cell. The red color indicates the known pathways. The details of EPHA2 pathway is shown in the figure

Analysis of published mouse LF cell data shows that, as expected, LF cell signaling pathways are very similar to LECs (Fig. 5). We have obtained 13,790 specific paths (i.e., 13.41% of total paths) after all filtering for mouse LF cell. Differences in the ranking of particular pathways provide indications of how these pathways are integrated in the transition from LECs to LF cells (e.g., EPHA2 in Figs 3, 4 and 5). Overall, these results show that the SPAGI R package can accurately identify and rank known, critically-important signaling pathways from the gene expression profiles of different cell and tissue types. As shown in Figs. 2 to 5, the SPAGI approach identifies each specific R, K and TF within each path and pathway, thereby enabling critical pathway-specific nodes to be identified, as well as critical nodes that interconnect between different pathways. Additionally, new candidate critical tissue regulators can be identified via the activity score ranking. For example, KREMEN1and KREMEN2 are known to regulate Wnt signaling pathways [35, 36], so these can be the potential active pathways for lens. Also PVRL3 is known to be associated for congenital ocular disease [37], so this can also be a potential active pathway for lens.

### Comparison of SPAGI analysis on species-specific vs combined PPI data

To determine the breadth of PPI data coverage within the mouse and human STRING datasets, we also performed the SPAGI analysis separately for the human and mouse query data – i.e., mouse samples compared only against the mouse STRING data and the human sample compared only against the human STRING data (see Additional File 1: Figures S1–4). From these results we see that a number of known pathways were identified for each sample. However, pathways known from previous studies to be important for particular cell types were not identified using this species-specific STRING analysis approach. For example, the ROR1 pathway was not identified in the human ROR1^+^ cell sample, despite ROR1 being critical for capturing this population of human LECs. Similarly, the EPHA2 pathway was not identified within the mouse LF cell samples, despite this being a key pathway that leads to disease (i.e., cataract) if disrupted [38]. Thus, combining the mouse and human PPI datasets prior to SPAGI analysis led to more biologically-relevant results for the query samples than obtained when using the human and mouse PPI data separately.

### Analysis of the SPAGI false positive rate via random expression level assignment

We investigated the false positive rate of the SPAGI method by randomly assigning gene expression data using both the mouse dental epithelial cell and mouse LEC gene expression data sets. First, we have randomly assigned the gene names amongst the gene expression values for each sample and then re-performed the SPAGI analyses as done for the original data. We then counted the number of highly ranked active pathways for each sample, and looked for identification of known pathways within the high ranked active pathways to investigate the SPAGI false discovery rate for known pathways from the randomly assigned expression data. Next we calculated the false positive rate for each sample utilizing the number of high ranked pathways of randomly assigned expression sample by dividing the number of high ranked pathways of original sample. We repeated this analysis 10 times for each sample and calculated the average false positive rate for each sample. The average false positive rate for mouse dental epithelial cell is 0.128 and for mouse LEC gene expression data is 0.022 (see Additional File 1: Tables S1 and S2).

### Analysis of the SPAGI false positive rate versus GO analysis

We also compared the performance of the SPAGI method with GO analysis method. The GO analysis was performed based on the unique set of molecules (i.e., Rs, Ks and TFs) from the original mouse dental epithelial cell and mouse LEC data. For comparison, we also performed GO analysis based on the same random assignment used to determine the SPAGI false discovery rate described above. Each GO analysis was performed separately using the online version of Enrichr [36], and captured all the results associated with biological process. These results were filtered to retain only the significant terms based on raw *p*-value and for signaling GO terms. Finally we searched for known pathways for each sample. Additional File 1: Table S3 shows the comparison results of the original cell samples, and Additional File 1: Tables S4 and S5 show the comparison results obtained using the randomly assigned cell samples. The results show that both the SPAGI and GO methods can identify almost all the known pathways for the original sample data, although the GO method did not identify the Cadherins pathways in the mouse LECs data. However, the results of the randomly assigned gene expression data showed that the false identification rate of known pathways by SPAGI was much smaller (0–0.2) than for the GO analysis method (0.4–1) (Additional File 1: Table S6).

## Discussion

In this manuscript we described a new bioinformatics method, SPAGI, that can simultaneously and comprehensively discover the set of active signaling pathways and their putative defined path structures. Our evaluation demonstrates that the SPAGI method can accurately identify known and biologically-relevant signaling pathways from multiple gene expression data sets across different tissue types, while providing specific detail of the molecular cascades involved in these pathways. The SPAGI method therefore provides capabilities not available with other current open-source software. While some pathway analysis software is commercially available (e.g, IPA), SPAGI provides a free and open-source approach that can routinely provide updated data through updates to the STRING database.

In addition to validation of the SPAGI method by comparison against known biology, the SPAGI approach was also validated by assessment of its false positive rate - both on its own and in comparison to the false positive rate obtained via GO analysis. The SPAGI approach identified few pathways when using randomly assigned gene expression data for the mouse dental epithelial cells (0.128) and mouse LECs (0.022). Moreover, the results of the randomly assign gene expression data showed the false positive rate was smaller for the SPAGI method (0–0.2) than the false positive rate obtained via the GO analysis method (0.4–1). These data provide strong support for SPAGI being both more sensitive and more specific than pathway identification via GO analysis alone.

To assess whether the SPAGI method is best applied to species-specific PPI data or combined/multi-species PPI data, were performed SPAGI analysis on both single species and combined species PPI data. While large numbers of pathways were identified via the single species analyses, some biologically-relevant pathways were not identified. This included the ROR1 receptor-mediated pathway not being identified via the human PPI data, and the EPHA2 pathway not being identified in the mouse LF cell data. As both these pathways appear to be important in their respective cell types [32, 38], SPAGI is currently best performed (i.e., identifies the largest number of biologically-relevant pathways) using the combined species PPI data.

It should be noted that as currently applied, the SPAGI method detects receptor-mediated signaling pathways. Modification of the SPAGI approach could be used to identify other cellular control mechanisms involving PPIs independent of TFs. Also, at this stage it is not clear whether the other pathways highly ranked by the activity score are truly active, as protein expression and protein activation state (e.g., via phosphorylation) within a tissue cannot be determined from gene expression data. Nonetheless, the breadth of data provided by SPAGI can provide specific testable hypotheses for cell biologists to guide functional genomic studies to identify critical regulators involved in health and disease. As such, more studies are required to investigate these pathways.

## Conclusions

The SPAGI method represents a new, interesting and open-source method to comprehensively identify important receptor-mediated signaling pathways from a gene expression data set. We have applied our method to four different gene expression data sets from three different cell types and shown that the SPAGI method correctly identified all the known signaling pathways for the cells, with low false discover rate and lower false discovery than using GO analysis alone. Our results suggest that SPAGI can be a useful approach to identify the potential active signaling pathways given a gene expression profile.

## Additional file

Additional File 1: **Figure S1.** The result of mouse embryonic dental epithelium cell at E13.5 with only the mouse PPI background pathway data. **Figure S2.** The result of newborn mouse lens epithelium cell with only the mouse PPI background pathway data. **Figure S3.** The result of human PSC-derived ROR1+ cell (lens epithelium cell-like) with only the human PPI background pathway data. **Figure S4.** The result of newborn mouse lens fiber cell with only the mouse PPI background pathway data. **Table S1.** False positive rate calculation for SPAGI method of randomly assigns gene expression values of new born mouse lens epithelial cell and mouse tooth epithelial cell at embryonic day E13.5. **Table S2.** SPAGI test result for randomly assign gene expression values of new born mouse lens epithelial cell and mouse tooth epithelial cell at embryonic day E13.5. **Table S3.** Identification of known pathways by SPAGI and GO analysis methods. **Table S4.** Summary of known pathways identification by SPAGI and GO methods for randomly assigns genes of mouse lens epithelial cell**. Table S5.** Summary of known pathways identification by SPAGI and GO methods of randomly assigns genes for mouse tooth epithelial cell. **Table S6.** False positive rate of SPAGI and GO analysis method for known pathways. (PDF 658 kb)

## Abbreviations

APN: Activity pathway network
K: Kinase
LEC: Lens epithelial cell
LF: Lens fiber
PPI: Protein-protein interaction
R: Receptor
SPAGI: Signaling pathway analysis for putative gene regulatory network identification
TF: Transcription factor
