# Supplementary material for: Identification of active signaling pathways by integrating gene expression and protein interaction data
Source: BMC Syst Biol. 2018 Dec 31;12(Suppl 9):120. doi: 10.1186/s12918-018-0655-x (PMC6311899; doi:10.1186/s12918-018-0655-x)
Supplement: Supplementary file 1 — Figure S1. The result of mouse embryonic dental epithelium cell at E13.5 with only the mouse PPI background pathway data. Figure S2. The result of newborn mouse lens epithelium cell with only the mouse PPI background pathway data. Figure S3. The result of human PSC-derived ROR1+ cell (lens epithelium cell-like) with only the human PPI background pathway data. Figure S4. The result of newborn mouse lens fiber cell with only the mouse PPI background pathway data. Table S1. False positive rate calculation for SPAGI method of randomly assigns gene expression values of new born mouse lens epithelial cell and mouse tooth epithelial cell at embryonic day E13.5. Table S2. SPAGI test result for randomly assign gene expression values of new born mouse lens epithelial cell and mouse tooth epithelial cell at embryonic day E13.5. Table S3. Identification of known pathways by SPAGI and GO analysis methods. Table S4. Summary of known pathways identification by SPAGI and GO methods for randomly assigns genes of mouse lens epithelial cell. Table S5. Summary of known pathways identification by SPAGI and GO methods of randomly assigns genes for mouse tooth epithelial cell. Table S6. False positive rate of SPAGI and GO analysis method for known pathways. (PDF 658 kb) [file 12918_2018_655_MOESM1_ESM.pdf]

Supplementary figures and tables

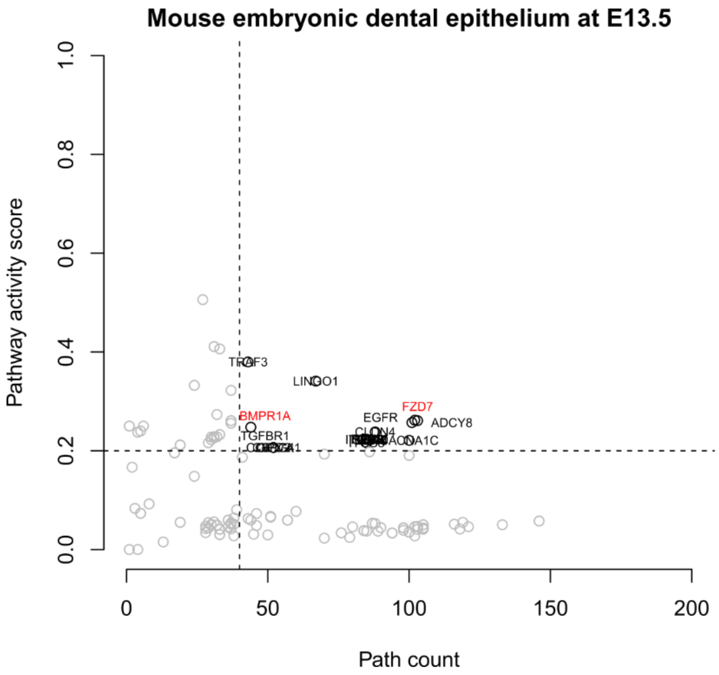

Figure S1. The result of mouse embryonic dental epithelium cell at E13.5 with only the mouse PPI background pathway data.

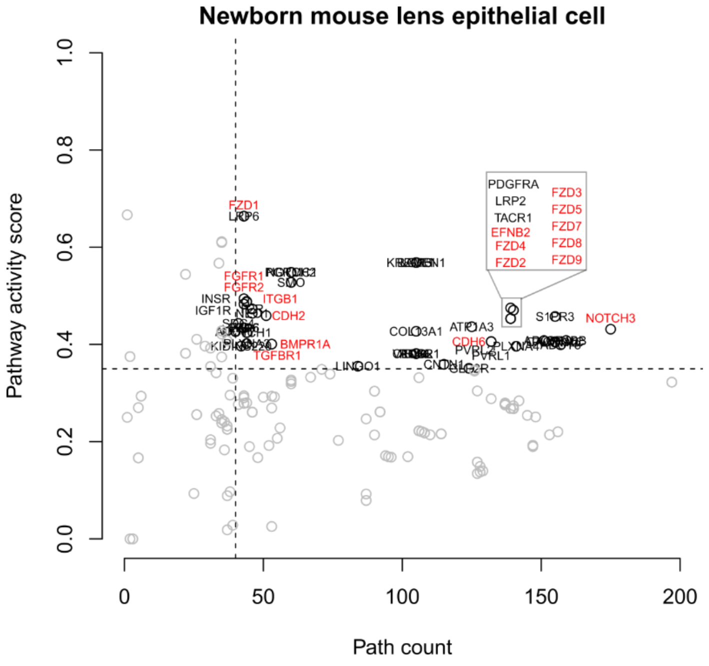

Figure S2. The result of newborn mouse lens epithelium cell with only the mouse PPI background pathway data.

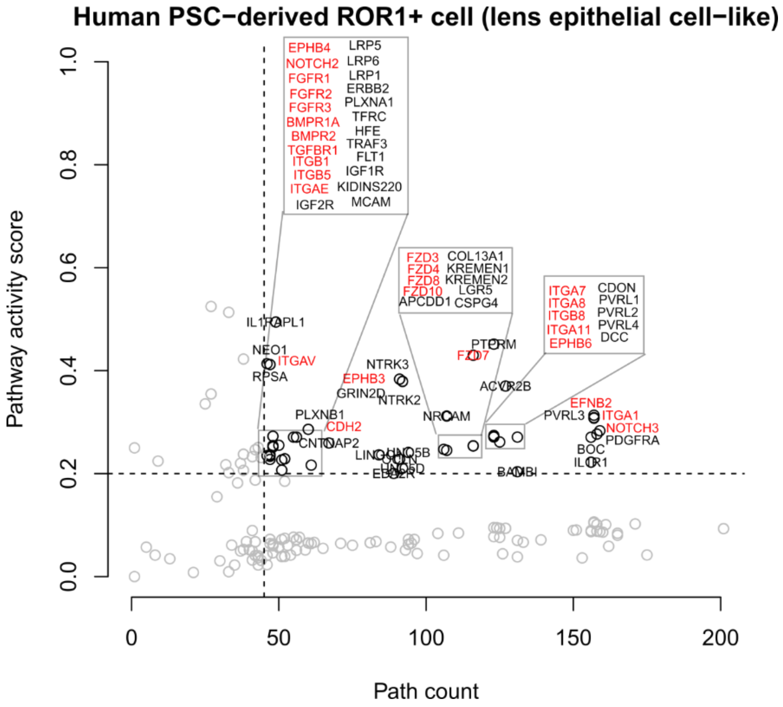

Figure S3. The result of human PSC-derived ROR1+ cell (lens epithelium cell-like) with only the human PPI background pathway data.

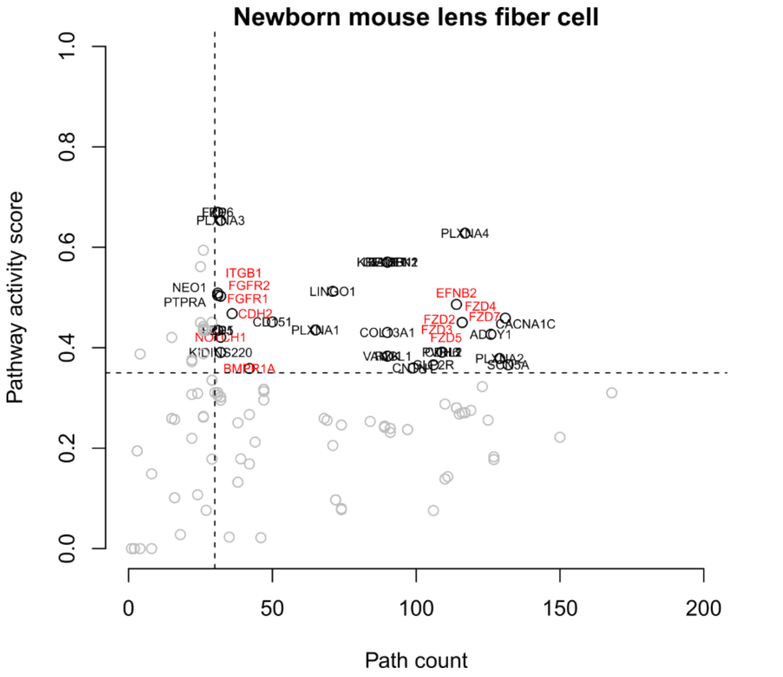

Figure S4. The result of newborn mouse lens fiber cell with only the mouse PPI background pathway data.

Table S1. False positive rate calculation for SPAGI method of randomly assigns gene expression values of new born mouse lens epithelial cell and mouse tooth epithelial cell at embryonic day E13.5

	New born mouse LEC		Mouse tooth epi at E13.5	
	# of high ranked pathways	False positive rate	# of high ranked pathways	False positive rate
**With original expression**	**66**		**21**	
Random assignment 1	6	0.09	0	0
Random assignment 2	0	0	7	0.33
Random assignment 3	1	0.02	0	0
Random assignment 4	0	0	8	0.38
Random assignment 5	0	0	0	0
Random assignment 6	0	0	0	0
Random assignment 7	0	0	3	0.14
Random assignment 8	7	0.11	9	0.43
Random assignment 9	0	0	0	0
Random assignment 10	0	0	0	0
	Total false positive rate	0.22	Total false positive rate	1.28
	**Average false positive rate**	**0.022**	**Average false positive rate**	**0.128**

Table S2. SPAGI test result for randomly assign gene expression values of new born mouse lens epithelial cell and mouse tooth epithelial cell at embryonic day E13.5

	New born mouse lens epithelial cell	Mouse tooth epithelial cell at embryonic day E13.5
**Random assignment 1**	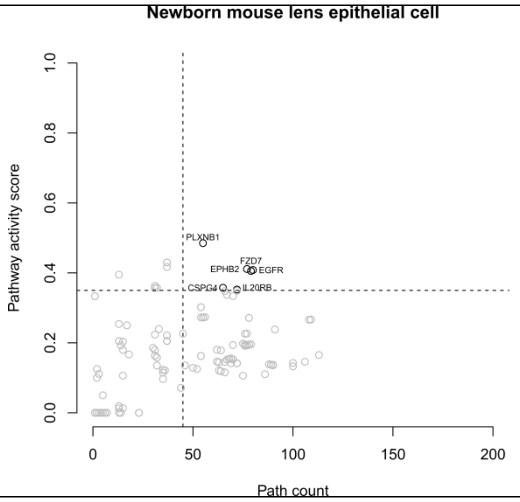	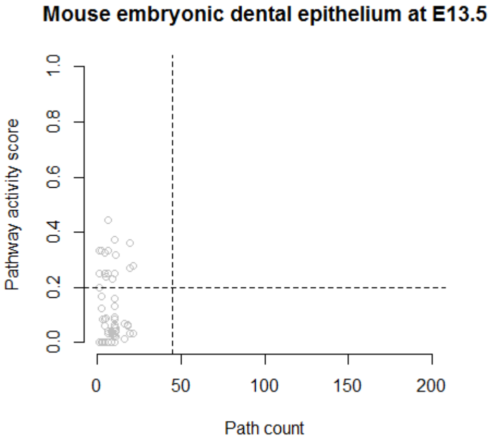

**Random assignment 2**	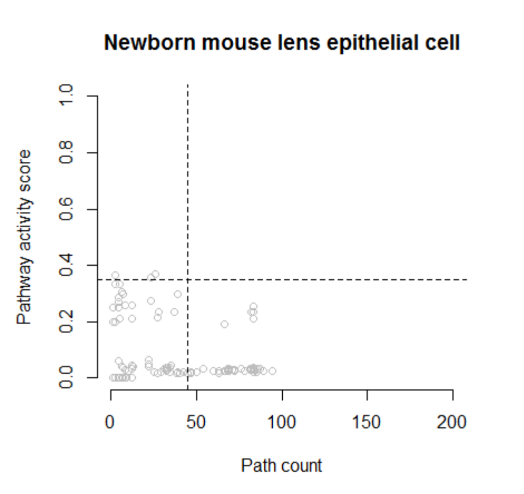	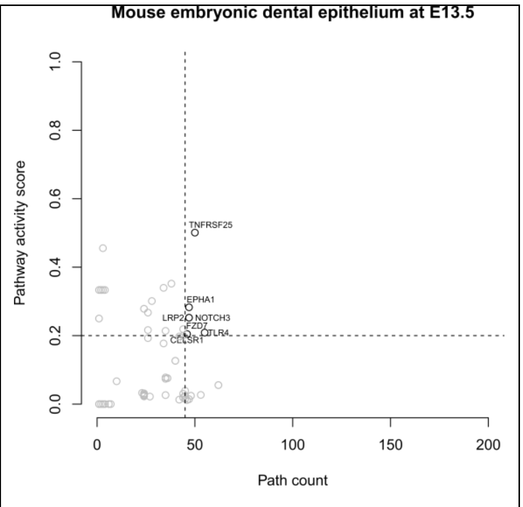
**Random assignment 3**	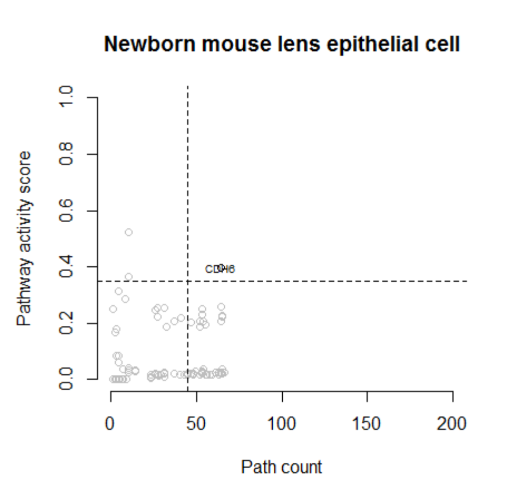	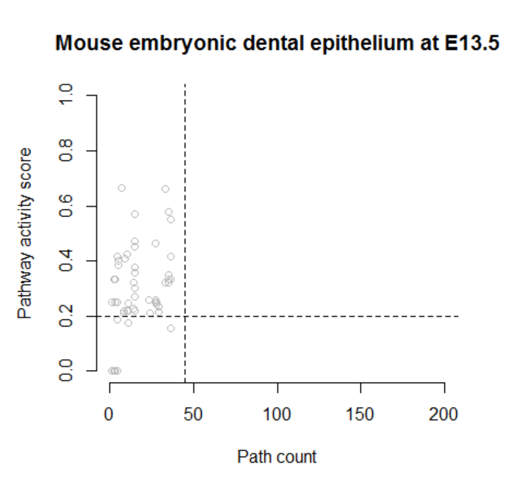
**Random assignment 4**	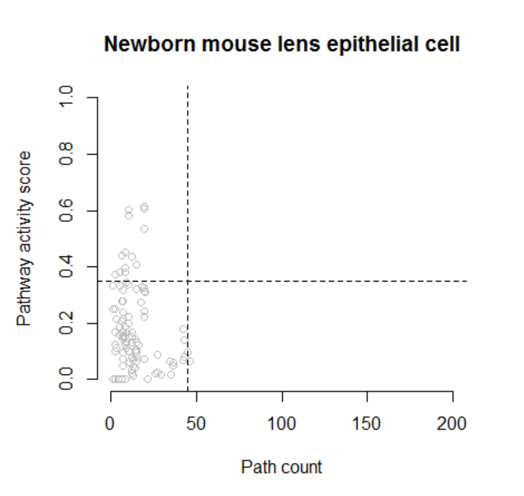	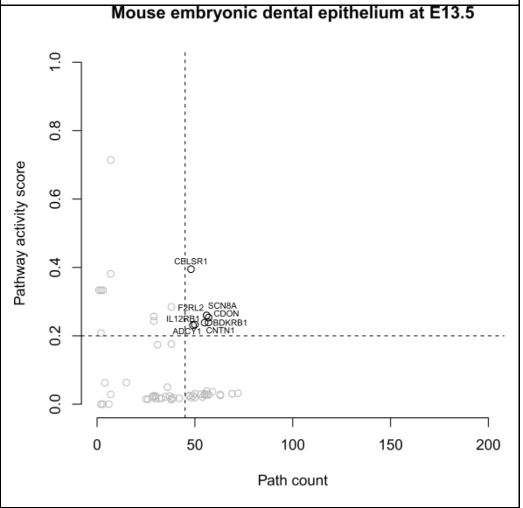

**Random assignment 5**	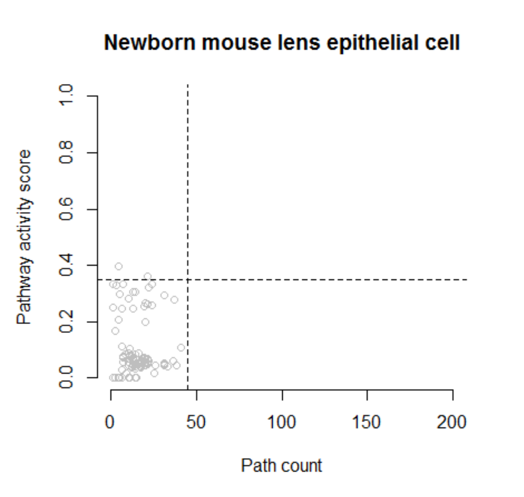	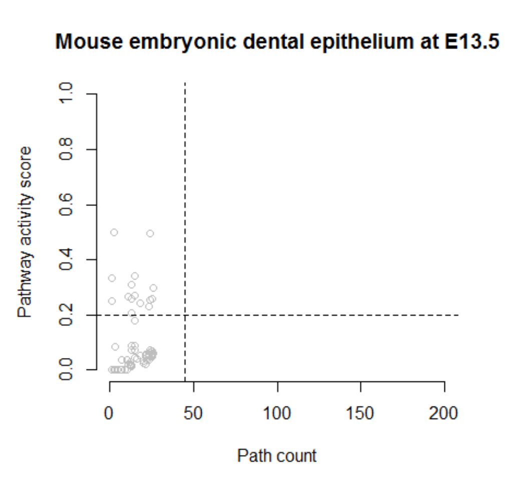
**Random assignment 6**	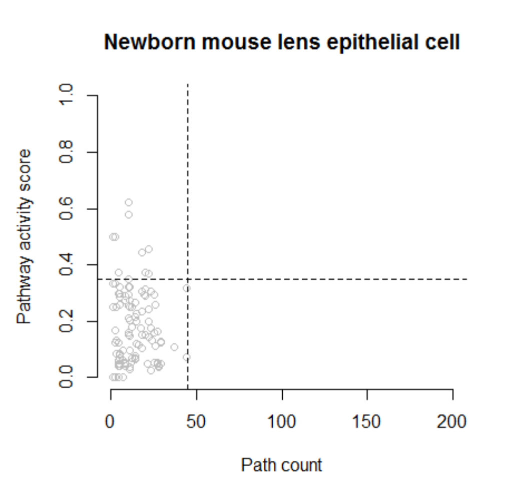	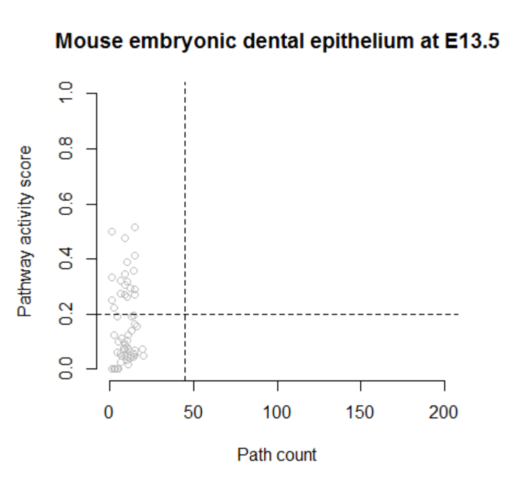
**Random assignment 7**	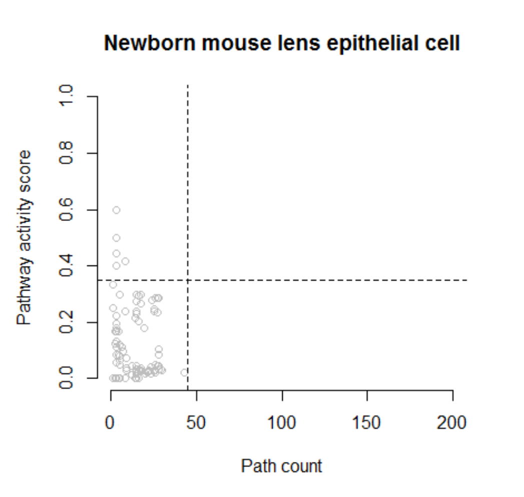	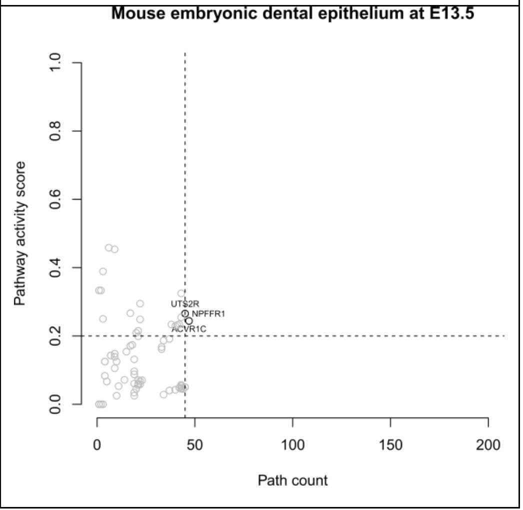

Random assignment 8

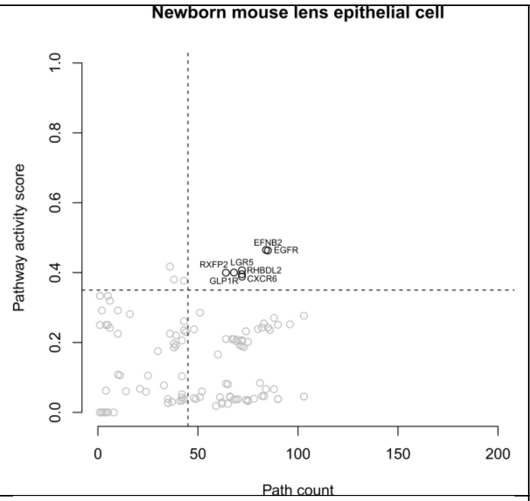

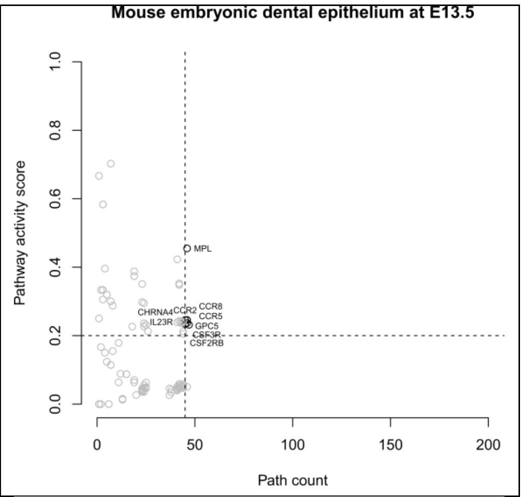

Random assignment 9

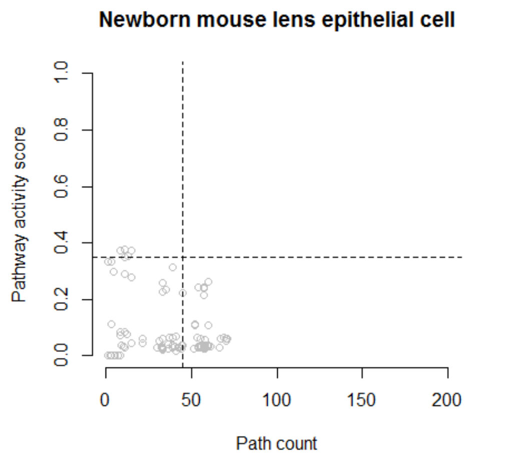

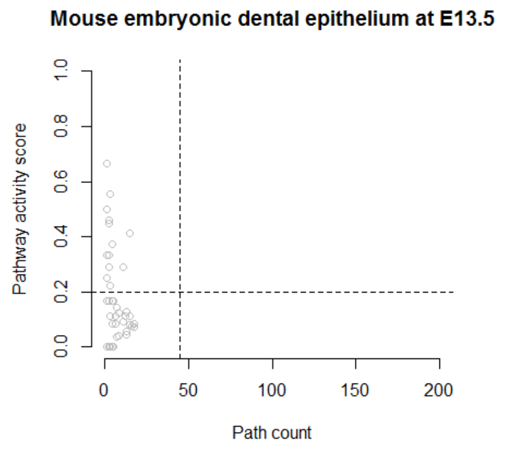

Random assignment 10

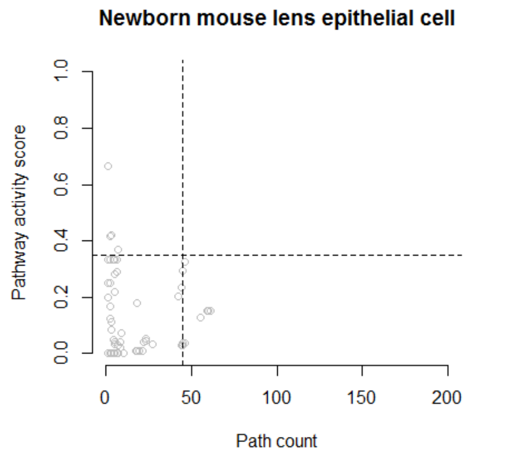

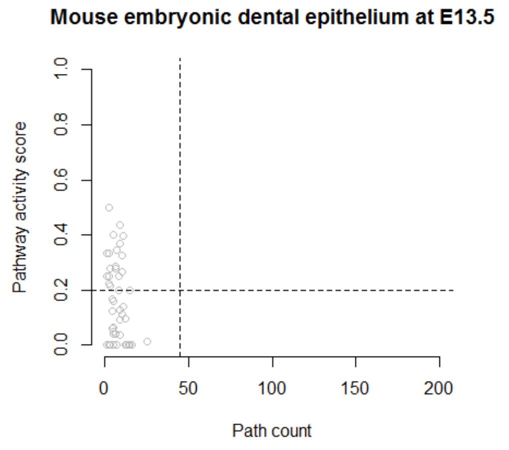

Table S3. Identification of known pathways by SPAGI and GO analysis methods

	BMP	Cadherins	EPH/Ephrin	FGF	Integrins	Notch	TGFB	Wnt
**Known for lens epithelial cell**	Y	Y	Y	Y	Y	Y	Y	Y
Identified by SPAGI	Y	Y	Y	Y	Y	Y	Y	Y
Identified by GO	Y	N	Y	Y	Y	Y	Y	Y
**Known for tooth epithelial cell**	Y							Y
Identified by SPAGI	Y							Y
Identified by GO	Y							Y

Table S4. Summary of known pathways identification by SPAGI and GO methods for randomly assigns genes of mouse lens epithelial cell

	BMP		Cadherins		EPH/Ephrin		FGF		Integrins		Notch		TGFB		Wnt	
	SPAGI	GO	SPAGI	GO	SPAGI	GO	SPAGI	GO	SPAGI	GO	SPAGI	GO	SPAGI	GO	SPAGI	GO
Random assignment 1	0	1	0	0	1	1	0	0	0	1	0	1	0	1	1	1
Random assignment 2	0	1	0	0	0	1	0	0	0	1	0	1	0	1	0	1
Random assignment 3	0	1	1	0	0	1	0	0	0	1	0	1	0	1	0	1
Random assignment 4	0	1	0	0	0	1	0	1	0	1	0	1	0	1	0	1
Random assignment 5	0	1	0	0	0	1	0	1	0	1	0	1	0	1	0	1
Random assignment 6	0	1	0	0	0	1	0	1	0	1	0	1	0	1	0	1
Random assignment 7	0	0	0	0	0	1	0	0	0	1	0	1	0	1	0	1
Random assignment 8	0	1	0	0	1	1	0	1	0	1	0	1	0	1	0	1
Random assignment 9	0	1	0	0	0	1	0	0	0	1	0	1	0	1	0	1
Random assignment 10	0	1	0	0	0	1	0	0	0	1	0	1	0	1	0	1
**Total**	**0**	**9**	**1**	**0**	**2**	**10**	**0**	**4**	**0**	**10**	**0**	**10**	**0**	**10**	**1**	**10**

Table S5. Summary of known pathways identification by SPAGI and GO methods of randomly assigns genes for mouse tooth epithelial cell

	BMP		Wnt (Fzd)	
	SPAGI	GO	SPAGI	GO
Random assignment 1	0	0	0	1
Random assignment 2	0	1	1	1
Random assignment 3	0	1	0	1
Random assignment 4	0	1	0	1
Random assignment 5	0	1	0	1
Random assignment 6	0	0	0	1
Random assignment 7	0	1	0	1
Random assignment 8	0	0	0	1
Random assignment 9	0	0	0	0
Random assignment 10	0	1	0	1
**Total**	**0**	**6**	**1**	**9**

Table S6. False positive rate of SPAGI and GO analysis method for known pathways

		BMP	Cadherins	EPH/Ephrin	FGF	Integrins	Notch	TGFB	Wnt
**Lens epithelial cell**	SPAGI	0	0.1	0.2	0	0	0	0	0.1
	GO	0.9	0	1	0.4	1	1	1	1
**Tooth epithelial cell**	SPAGI	0							0.1
	GO	0.6							0.9
